# Influence of opioid analgesia type on circulating tumor cells in open colorectal cancer surgery (POACC-1): study protocol for a prospective randomized multicenter controlled trial

**DOI:** 10.1186/s12871-023-02007-1

**Published:** 2023-02-28

**Authors:** Emil Berta, Josef Srovnal, Petr Dytrych, Jan Bruthans, Jitka Ulrichova, Petr Prasil, Lubomir Vecera, Tomas Gabrhelik, Benjamin Tolmaci, Josef Dusa, Jan Maca, Michelle Mazancova, Filip Haiduk, Martin Kutej, Peter Ihnat, Pavel Michalek, Marian Hajduch

**Affiliations:** 1 Institute of Molecular and Translational Medicine, Faculty of Medicine and Dentistry, Palacky, University and University Hospital in Olomouc, Olomouc, Czech Republic; 2 Ringerike Hospital, VVHF, Honefoss, Norway; 3 Department of Anesthesiology and Intensive Medicine, General University Hospital and First Medical Faculty of the Charles University, Prague, Czech Republic; 4 1st Department of Surgery - Department of Abdominal, Thoracic Surgery and Traumatology, First Faculty of Medicine, Charles University and General University Hospital, Prague, Czech Republic; 5 Department of Anesthesiology, Landesklinikum Amstetten, Amstetten, Austria; 6 Department of Anesthesiology and Intensive Medicine, Tomas Bata Regional Hospital in Zlin, Zlin, Czech Republic; 7 Department of Paediatric Anaesthesiology and Intensive Care Medicine, Medical Faculty of Masaryk University, University Hospital Brno, Brno, Czech Republic; 8 Department of Anesthesiology and Intensive Medicine, Faculty of Medicine and Dentistry, Palacky University and University Hospital in Olomouc, Olomouc, Czech Republic; 9 Department of Surgery, Tomas Bata Regional Hospital in Zlin, Zlin, Czech Republic; 10 Department of Oncology, Faculty of Medicine and Dentistry, Palacky University and University Hospital in Olomouc, Olomouc, Czech Republic; 11 Department of Anesthesiology and Intensive Care Medicine, University Hospital Ostrava, Ostrava, Czech Republic; 12 University of Ostrava, Institute of Physiology and Pathophysiology, Faculty of Medicine, Ostrava, Czech Republic; 13 Department of Intensive Medicine, University of Ostrava, Department of Emergency Medicine and Forensic Studies, Faculty of Medicine, Ostrava, Czech Republic; 14 Department of Anesthesiology and Intensive Care Medicine, University in Brno, Faculty of Medicine, Masaryk University, Brno, Czech Republic; 15 Department of Surgery, University Hospital Ostrava, Ostrava, Czech Republic; 16 Department of Surgical Studies, Faculty of Medicine, University of Ostrava, Ostrava, Czech Republic

**Keywords:** Epidural, Morphine, Piritramide, Perioperative analgesia, Colorectal cancer, Cancer recurrence, Circulating tumor cells, Colorectal surgery

## Abstract

**Background:**

Opioids and epidural analgesia are a mainstay of perioperative analgesia but their influence on cancer recurrence remains unclear. Based on retrospective data, we found that cancer recurrence following colorectal cancer surgery correlates with the number of circulating tumor cells (CTCs) in the early postoperative period. Also, morphine- but not piritramide-based postoperative analgesia increases the presence of CTCs and shortens cancer-specific survival. The influence of epidural analgesia on CTCs has not been studied yet.

**Methods:**

We intend to enroll 120 patients in four centers in this prospective randomized controlled trial. The study protocol has been approved by Ethics Committees in all participating centers. Patients undergoing radical open colorectal cancer surgery are randomized into epidural, morphine, and piritramide groups for perioperative analgesia. The primary outcome is the difference in the number of CTCs in the peripheral blood before surgery, on the second postoperative day, and 2–4 weeks after surgery. The number of CTCs is measured using molecular biology methods. Perioperative care is standardized, and relevant data is recorded. A secondary outcome, if feasible, would be the expression and activity of various receptor subtypes in cancer tissue. We intend to perform a 5-year follow-up with regard to metastasis development.

**Discussion:**

The mode of perioperative analgesia favorably affecting cancer recurrence would decrease morbidity/mortality. To identify such techniques, trials with long-term follow-up periods seem suboptimal. Given complex oncological therapeutic strategies, such trials likely disable the separation of perioperative analgesia effects from other factors. We believe that early postoperative CTCs presence/dynamics may serve as a sensitive marker of various perioperative interventions´ influences on cancer recurrence. Importantly, it is unbiased to the influence of long-term factors and minimally invasive. Analysis of opioid/cannabinoid receptor subtypes in cancer tissue would improve understanding of underlying mechanisms and promote personalization of treatment. We are not aware of any similar ongoing studies.

**Trial registration number:**

NCT03700411, registration date: October 3, 2018. Study status: recruiting.

## Background

Colorectal cancer (CRC) is the second most common cause of cancer-related deaths in Europe. Despite the progress in its early detection and adjuvant treatment, metastases are the leading cause of death in this group of patients. Although the development of postoperative metastases is a multifactorial process, it has been suggested that the mode of perioperative analgesia (PA) may play an important role in cancer recurrence (CR) [1, 2]. It has been proposed that avoiding opioids (particularly morphine) and employing regional analgesia techniques may have favorable effects on CR [1]. Such effects, if present, are probably attributable to less suppression of antitumor immune defenses due to:

1. Better analgesia leading to a less pronounced perioperative stress reaction [1] which preserves anticancer immunity (particularly natural killer cells’ cytotoxicity). Such effects of epidural analgesia have been observed in open colectomy [3] and other major abdominal surgeries [4].
2. Reduced exposition to volatile and intravenous anesthetics [1].
3. Reduced exposition to opioids, especially morphine which exhibits negative effects on anticancer immunity and increases cancer cell proliferation, tumor progression [5, 6], and CR [2, 5, 7–10]. However, data on angiogenesis [8, 11–14] and cancer cell invasion [15, 16] are conflicting and some studies even show favorable immunomodulation [8].
4. Positive effects of local anesthetics (cytotoxic and anti-proliferative). Indeed, promising results of in vitro [17] and animal studies showed that regional anesthesia decreases the risk of metastasis [5, 18, 19].

During the last 15 years, several retrospective and post-hoc analyses yielded conflicting results regarding the effects of PA on cancer recurrence following CRC surgery. Christopherson et al. observed that epidural analgesia was associated with improved survival in CRC patients without metastases. However the effect lasted for only 1.46 years postoperatively, may have been caused by a reduction in early postoperative adverse events and the data come from 1992–1994 rendering them less relevant for current treatment standards [20]. In post-hoc analysis, Gottschalk et al. observed the potential benefits of epidural analgesia but limited only to patients > 64 years [21]. Gupta et al. observed a reduction in all-cause mortality after rectal but not colon cancer surgery using epidural analgesia but the mean follow-up was short (2.6 years) and actual causes of death were unknown [22]. More recently, Zimmitti et al. showed that epidural analgesia improved recurrence-free survival and overall survival in patients undergoing hepatic resection for liver metastases despite significantly longer operative times compared to the control group [23]. Also, Vogelaar et al. observed improved five-year survival in the epidural analgesia group of CRC surgical patients and this effect was even more significant in a subgroup of patients > 80 years [24]. In an in vitro study, Xu et al. found that the serum from colon cancer patients receiving propofol and epidural analgesia inhibited proliferation and invasion of LoVo colon cancer cells and induced apoptosis more than in patients receiving sevoflurane anesthesia with opioid analgesia [25]. This finding shows that type of anesthesia and PA may affect the immune response and/or cancer cell biology potentially leading to a lower risk of metastasis in CRC.

On the other hand, in a large study in 42 151 patients undergoing colectomy for CRC, Cummings et al. found no improvement in CR in the epidural analgesia group (despite the fact that patients receiving epidural analgesia had better 5-year survival) [26]. Recently, Wu et al. observed no favorable effect of epidural analgesia on CR following curative colon cancer surgery in a single-center trial [27]. Likewise, Hasselager et al. found no CR benefit when analyzing 11 618 patients undergoing curative CRC surgery [28]. However, in both these trials, both open and minimally invasive surgery patients were included and patient recruitment stretched over long periods of time. The latter trial included also rectal cancer patients who received epidural analgesia more often. Also, Falk et al. published two negative findings in CRC surgery patients—in a retrospective analysis of more than 5700 patients undergoing both open and minimally invasive curative surgery, no survival benefit was observed in the epidural analgesia group at 30 days, 90 days, and 3 years [29]. In the only available prospective randomized multicenter trial comparing epidural analgesia and morphine-based analgesia in a mixed open and minimally invasive surgery population, Falk et al. found no statistically significant difference in disease-free survival [30]. However, a 7% improvement in disease-free survival was detected in the epidural analgesia group but the trial was underpowered to reach statistical significance. Moreover, the trial had to be stopped prematurely due to recruitment problems related to the increasing number of minimally invasive surgeries.

Clearly, a major limitation of the clinical trials studying CR is the long follow-up needed to assess survival outcomes. Effects of complex (and changing) oncological therapeutic strategies employed in colorectal and other cancers complicate the separation of PA effects from those of other interventions. And, adding to the complexity of various perioperative factors, anesthetics such as sevoflurane and propofol may also promote anticancer activity through the modulation of microRNA molecules that control post-transcriptional gene regulation [31]. Thus, based on the available data, it is difficult to recommend an optimal type of PA (and anesthesia) for CRC patients. Prospective trials employing methods enabling the assessment of PA effects independently of other factors are needed.

### Circulating tumor cells as the markers of CR

Following radical surgery, metastatic potential persists in the form of circulating tumor cells (CTCs) and disseminated tumor cells (DTCs) [32]. These cells may develop into macroscopic metastases [33, 34]. The process is likely accelerated by postoperative immunosuppression lasting for several days [35]. Interactions between the immune system and cancer cells are complex involving non-specific (natural killer cells, macrophages, neutrophils) as well as antigen-specific immune cells [32]. Both cancer and immune system cells are influenced by analgesic techniques [2].

We propose that CTCs presence and dynamics may serve as a sensitive surrogate biomarker of the effects of various perioperative factors (including analgesic techniques) on CR and survival. In CRC patients, it has been shown that CTCs presence during the first postoperative weeks is an independent negative prognostic factor [36, 37]. Our study group also found that morphine- but not piritramide-based analgesia increases CTCs presence [38], which may be attributable to different effects of piritramide and morphine on cell membrane receptors. Therefore, we believe that it is necessary to study the effects of PA on the presence of CTCs as well as on membrane receptors expressed on tumor tissue cells in order to personalize for the right patient at the right time. To the best of our knowledge, no similar studies are currently ongoing. Likewise, we found no studies on piritramide versus morphine effects on CTCs presence and/or CR.

We are aware that the evaluation of CTCs presence and dynamics in the perioperative period is not equivalent to clinical CR parameters, considering the importance of long-term factors involved (adjuvant therapy, lifestyle, and comorbidities). However, we believe that it can serve as a reasonably sensitive laboratory biomarker of the effects of perioperative factors on CR, unbiased to the influence of long-term factors. This approach should simplify the identification of techniques that support antimetastatic immune responses and decrease CTCs/DTCs numbers and/or invasivity, mainly in the vulnerable postoperative period. Such techniques should diminish the risk of CR, potentially reducing morbidity and improving survival without additional expenses or burdens for the patients.

## Methods

The study protocol has been prepared using the SPIRIT 2013 Statement guidelines [39] and in full concordance with the Declaration of Helsinki.

### Project objectives

The aim of the project is to identify optimal analgesia techniques for CR prevention in open radical CRC surgery. Also, the aim is to elucidate the mechanisms of metastasis with regard to the expression and activity of various receptor subtypes in cancer tissues and the effects of morphine and piritramide on these receptors. Therefore, we intend to perform a prospective, randomized, controlled, multicenter trial comparing the effects of epidural, morphine- and piritramide-based analgesia on CTCs presence and dynamics in radical open CRC surgery. Correlations between the type of PA and the number of CTCs in the early postoperative period will be studied. We formulated the following working hypotheses:

- Different methods of analgesia affect CTCs presence and metastasis following CRC surgery.
- In CRC patients, the number of CTCs in the early postoperative period correlates positively with metastasis and negatively with survival.
- Epidural analgesia has favorable effects on CTCs presence and metastasis compared with morphine- and piritramide-based PA.
- Piritramide-based PA has favorable effects on CTCs presence and metastasis compared with morphine-based PA.

### Primary outcome

The difference between the number of CTCs prior to surgery and 2—4 weeks after the surgery.

### Secondary outcomes

Pain intensity will be assessed in regular intervals (4 h) during the first 72 postoperative hours using the Numerical Rating Scale (NRS). Other secondary outcomes include hemodynamic stability, the incidence of ileus, and incidence of postoperative nausea and vomiting.

The last secondary outcome is the expression and activity of various receptor subtypes in cancer tissue.

### Inclusion criteria

- Patients indicated for open radical CRC surgery
- Both genders – males and females
- Adults – aged > 18 years
- Capabilities to understand and sign the informed consent

### Exclusion criteria

- Intolerance or allergy to study drugs
- A history of previous colorectal surgery
- Active neoadjuvant therapy prior to enrollment
- Contraindications to epidural analgesia
- Another malignancy not in permanent remission
- Chronic opioid medication or opioid administration within 7 days preoperatively
- Immunosuppressive or corticosteroid therapy
- Surgery within 30 days preoperatively (except for minor procedures)
- Chronic or acute infections

### Clinical trial design

**Fig. 1:**
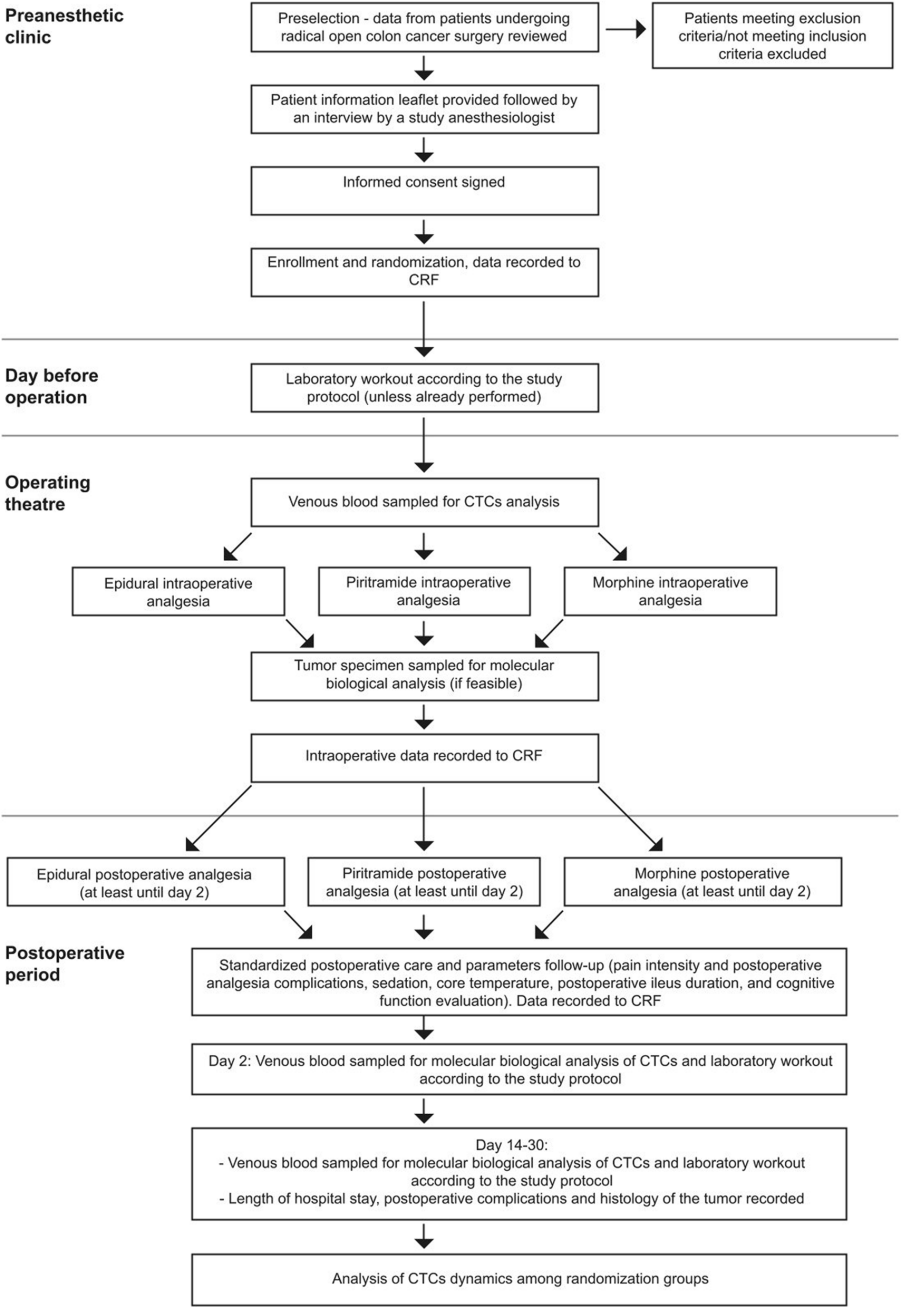
Study flowchart

The design of the clinical trial is described in detail in Fig. 1 representing the flowchart of the study.

### Sample size selection

Power analysis and sample size calculation stem from our prospective pilot data in 57 patients (17 patients with morphine, 19 patients with epidural, and 21 patients with piritramide analgesia). In this pilot study, we found a significant reduction in the number of CEA-mRNA positive CTCs (CEA = carcinoembryonic antigen) in systemic blood in 35% of piritramide- versus morphine-receiving patients. To confirm this effect, we expect a 35% decrease in CTCs in systemic blood in this ongoing study. We aim to demonstrate the effect in the epidural/piritramide analgesia groups at the level of 20%. Based on the power of 0.8 and the first type error of 0.05, we calculated the minimum number of subjects in the group to be 37. In order to compensate for a potential dropout of 10%, we decided to enroll minimum 40 patients per group, i.e. minimum 120 patients in total.

### Recruitment and randomization

Recruitment of patients undergoing radical open colon cancer surgery started on January 7, 2019 in three Czech hospitals (2 university and 1 regional). Patients fulfilling inclusion criteria are enrolled by the study anesthesiologist at the pre-anesthetic clinic and randomized into three arms according to the method of PA: 1. Continuous thoracic epidural analgesia, 2. Piritramide analgesia, and 3. Morphine analgesia. Randomization is performed using a computer-generated list with sequentially numbered containers (by a person not involved in the statistical analysis of the data). Demographic data, co-morbidities, cognitive function and physical status evaluations, chronic medication, preoperative oncologic therapy, laboratory test results, ASA status, and premedication are recorded into the study charts before surgery.

Blinding of the patients and operators is not possible due to clearly different types of intervention. The person performing data analysis will be blinded.

### Perioperative period

Patients are administered 400 ml/50 g carbohydrate pre-surgery drink, premedicated and anesthetized according to the standardized protocol which defines: perioperative medication, fluid therapy, hemodynamic goals and management, diuresis, core temperature, transfusion threshold, and postoperative nausea, and vomiting prevention. The protocol also defines analgesia corresponding with randomization (including a detailed standard of care for thoracic epidural analgesia). All the data are recorded including the length of anesthesia and surgery, and a brief description of the type of surgery and observed radicality.

Postoperative care is standardized including postoperative analgesia corresponding with randomization, hemodynamic optimization, fluid therapy and transfusions, glycemic control, and postoperative nausea and vomiting management.

**Fig. 2:**
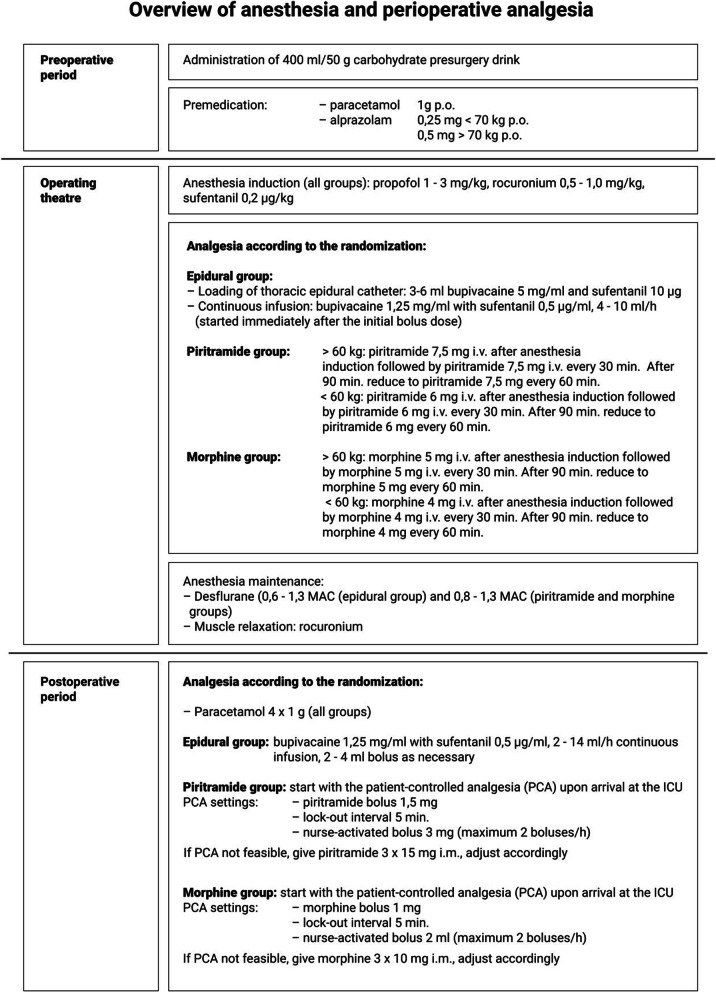
Detailed protocol of perioperative management

A detailed description of perioperative and postoperative management in all three arms of the study is displayed in Fig. 2. The patients with insufficient pain relief on the protocol medication will be provided rescue multimodal analgesia with a strong opioid and will be excluded from the final analysis of the CTCs.

All the data are recorded to the patient´s electronic CRF, using academic cloud operated software ClinData (https://clindata.imtm.cz), including pain intensity (using the Numerical Rating Scale) and sedation (using the Richmond Agitation-Sedation Scale), adverse effects and complications directly associated with PA, postoperative nausea and vomiting, core temperature, duration of postoperative ileus, laboratory tests, and cognitive function evaluation.

### Data monitoring

Data and the results of the trial will be audited and evaluated by the grant provider with its independent assessors. A trial progress report is submitted annually to the grant provider. The trial may be interrupted or terminated by the Ethical Committees of the institutions involved in the study or by the grant provider if there is a severe violation in the conduct of the trial or obvious harm to the participants. Adverse events are monitored at each site and immediately reported to the Ethical Committee and the grant provider. Potential protocol amendments are processed according to the Helsinki declaration.

### Sample collection, transport, and molecular biology analysis

In total, three samples of 9 ml of peripheral blood are collected into stabilization tubes (Streck cell-free DNA BC) from each patient: prior to anesthesia induction, on day 2, and on day 14–30. The amount of blood collected corresponds with other studies utilizing CytoTrack technology [40, 41]. We have chosen the sampling from the peripheral vein because it would not be possible to establish central venous access weeks after the surgical procedure. Stabilized samples are transported by express post/parcel service from participating hospitals to the Institute of Molecular and Translational Medicine (IMTM) on daily basis.

After delivery, blood samples will be immediately processed. After centrifugation at 2500 g for 15 min, the buffy coats will be further purified and stabilized using FACS lysing solution (BD Biosciences, San Jose, CA, USA) and subsequently stained with Cytotrack Reagent Kit (2C A/S, Copenhagen, Denmark) containing DAPI and anti-EpCAM, anti-panCK and anti-CD45 fluorescent- labeled antibodies. The immunostained cells will be applied on glass cytodiscs and scanned using an automatic fluorescent microscope CytoTrack CT11 (2C A/S, Copenhagen, Denmark). The CTCs number will be based on cell morphology, nuclei presence, and antibody expression (CK20 + , EpCAM + , CD45-) according to general recommendations.

The number of single CTCs and CTCs clusters is analyzed within each sample. If feasible, also the expression and activity of various receptor subtypes in cancer tissue would be analyzed.

### Methods of data stewardship and analysis

A transparent database for safe and efficient clinical data collection and stewardship was established within the ClinData software developed and deployed by the IMTM. The ClinData is open to all clinical centers for online management of the trial and recording of clinical and laboratory data. The data are converted into numeric codes for anonymization and follow-up processing.

Statistical analysis will be performed by an experienced biostatistician working full-time at the IMTM. Analysis of covariance (ANCOVA), repeated ANOVA tests or a non-parametric approach (Wilcoxon test, Kruskal–Wallis test) will be used to assess differences between respective groups with regard to changes in the number of CTCs before and after surgery. Cut-off values for the detection of CTCs and receptor expressions will be established using bootstrap methods. As feasible, the Kaplan Meier method will be used to analyze overall and/or disease-free survival and the log-rank test will be used to assess differences between respective groups. Alternatively, Cox regression analysis will be employed.

### Follow-up

A. Short-term (2–4 weeks after surgery): In all centers, sampling of peripheral venous blood is performed for the purpose of molecular analyses. Other standard laboratory tests including concentrations of tumor markers are performed. The length of hospital stay, postoperative complications as well as histology of the tumor are recorded.

B. Long-term (up to 5 years): We intend to follow the patients up beyond the duration of this project with regard to metastasis development according to current standards. Disease-free survival and cancer-specific and/or overall survival will be recorded.

### Dissemination

Study results will be disseminated through posters/lectures at regional and international conferences. We intend to publish a scientific manuscript in a peer-reviewed journal.

## Data Availability

The datasets generated and/or analyzed during the current study will be available at the: https://figshare.com following the completion of the trial. Model consent form for the participants is provided in Appendix 1.

## Abbreviations

ANOVA: Analysis of Variance
ASA: American Society of Anesthesiologists
CD45: Cluster of differentiation 45
CEA: Carcinoembryonic antigen
CK20: Cytokeratin 20
CR: Cancer Recurrence
CRC: Colorectal Cancer
CTCs: Circulating Tumor Cells
DAPI: 4’,6-Diamidino-2-phenylindole
DTCs: Disseminated Tumor Cells
EpCAM: Epithelial Cellular Adhesion Molecule
IMTM: Institute of Molecular and Translational Medicine
PA: Perioperative Analgesia
SPIRIT: Standard Protocol Items: Recommendations for Interventional Trials
